# Modeling maize aflatoxins and fumonisins in a Tanzanian smallholder system: Accounting for diverse risk factors improves mycotoxin models

**DOI:** 10.1371/journal.pone.0316457

**Published:** 2025-01-13

**Authors:** William Stafstrom, Francis Ngure, John Mshanga, Henry Wells, Rebecca J. Nelson, John Mischler

**Affiliations:** 1 School of Integrative Plant Science, Cornell University, Ithaca, NY, United States of America; 2 Independent Research Consultant, Mycotoxins Mitigation and Child Stunting Research Trial, Arusha Tanzania & Nairobi, Limuru, Kenya; 3 Division of Nutritional Sciences, Cornell University, Ithaca, NY, United States of America; 4 Department of Food Sciences and Biotechnology School of Life Sciences and Bioengineering, The Nelson Mandela African Institution of Science and Technology (NM-AIST), Arusha, Tanzania; 5 Sustainability and Environmental Education, Goshen College, Goshen, IN, United States of America; Universidade Federal do Amazonas, BRAZIL

## Abstract

Human exposure to mycotoxins is common and often severe in underregulated maize-based food systems. This study explored how monitoring of these systems could help to identify when and where outbreaks occur and inform potential mitigation efforts. Within a maize smallholder system in Kongwa District, Tanzania, we performed two food surveys of mycotoxin contamination at local grain mills, documenting high levels of aflatoxins and fumonisins in maize destined for human consumption. A farmer questionnaire documented diverse pre-harvest and post-harvest practices among smallholder farmers. We modeled maize aflatoxins and fumonisins as a function of diverse indicators of mycotoxin risk based on survey data, high-resolution geospatial environmental data (normalized difference vegetation index and soil quality), and proximal near-infrared spectroscopy. Interestingly, mixed linear models revealed that all data types explained some portion of variance in aflatoxin and fumonisin concentrations. Including all covariates, 2015 models explained 27.6% and 20.6% of variation in aflatoxin and fumonisin, and 2019 models explained 39.4% and 40.0% of variation in aflatoxin and fumonisin. This study demonstrates the value of using low-cost risk factors to model mycotoxins and provides a framework for designing and implementing mycotoxin monitoring within smallholder settings.

## Introduction

Ensuring safe and secure food systems is a global priority, and mycotoxin contamination is a significant obstacle to this goal. Mycotoxins are secondary metabolites produced by fungal plant pathogens that are toxic to humans and livestock. The negative effects caused by trace amounts of mycotoxins in staple foods are a significant challenge to food security, causing economic losses and burdens on public health [1, 2]. Globally, mycotoxins exceed regulatory limits in an estimated 25% of foods [3]. High-income countries employ resource-intensive regulatory structures and repurpose or eliminate foods exceeding established mycotoxin standards. These systems are largely effective but incur significant economic costs; in the United States of America, crop losses and mitigation efforts cost the economy an estimated $1.5 billion annually [4].

In resource-limited contexts, regulatory enforcement of mycotoxins is generally weak. The lack of effective regulation in many mycotoxin-prone environments means that chronic and episodic exposure to high levels of mycotoxins causes severe public health consequences. Liver cancer is associated with chronic aflatoxin consumption, *in utero* aflatoxin exposure increases rates of growth faltering, childhood stunting is associated with the presence fumonisin and aflatoxin in baby foods, and outbreaks of acute aflatoxicosis in Kenya and Tanzania have resulted in hundreds of deaths [5–12]. Frequently, one of the primary sources of mycotoxin-contaminated food is maize.

Maize is a key staple crop across many parts of sub-Saharan Africa (SSA) such as Tanzania, where it accounts for approximately forty percent of daily calories [13]. In Tanzania, milled maize is typically consumed as *ugali*, a stiff porridge, or it is combined with other milled grains and groundnuts to create a composite flour, *lishe*, that is used to make *uji*, a thinner porridge prepared primarily for children [14]. Consumption of *ugali* and *uji* exposes children and adults to numerous mycotoxins [15, 16]. Numerous mycotoxin-producing fungi infect and contaminate maize grain including, aflatoxin-producing *Aspergillus flavus* and *A*. *parasiticus*, fumonisin-producing *Fusarium verticillioides* and *F*. *proliferatum*, and deoxynivalenol-producing *F*. *graminearum* [17, 18]. Naturally occurring aflatoxins are classified as a Group 1 human carcinogen, and the most prevalent and toxic form of fumonisin, Fumonisin B1, is a Group 2B possible human carcinogen [19]. Maize is more susceptible to aflatoxin and fumonisin contamination than small grains like sorghums and millets [20, 21]. Mycotoxigenic fungi are adapted to different environments, but co-occurrence is frequently observed, particularly in warm and dry areas of SSA where both *A*. *flavus* and *F*. *verticillioides* thrive and produce aflatoxins and fumonisins, respectively [22, 23].

Maize is processed in SSA at a range of scales, from large-scale industrialized operations to small rural local mills [24]. Across most of SSA, mycotoxin regulations are largely unenforced, especially in rural subsistence contexts [25]. Smallholder farms are important maize producers in SSA, and these farms can be subsistence-only operations or, more commonly, they also interact with markets as buyers and/or sellers [26]. Smallholder farm systems are typically low-input and rainfed, which makes them more vulnerable to abiotic stressors like water and nutrient deficiencies [27]. With climate change, extreme weather events like drought are predicted (and observed) to be increasingly common in SSA and are projected to reduce maize yields and increase the risk of mycotoxin contamination [28–31].

Environmental and cultural factors intersect to make maize consumers in smallholder systems especially vulnerable to mycotoxin exposure [32]. Extreme hot and dry weather during the growing season stresses developing maize plants, making them more susceptible to infection by mycotoxigenic fungi, and thus at increased risk of mycotoxin contamination [33, 34]. Hotter weather provides the optimal growth temperatures for these *A*. *flavus* (32°C) and *F*. *verticillioides* (30°C) [35–37]. Also, hot and dry weather during flowering and grain fill periods of maize development facilitates opportunistic *A*. *flavus* infection and is associated with increased aflatoxin contamination, while warm and wet conditions later in the season provide an ideal environment for established fungal pathogens to grow [38]. Warmer temperatures during flowering were also associated with increased fumonisin contamination in maize [39]. Developing the capacity to predict mycotoxin accumulation in these production systems requires integrating knowledge of geospatial mycotoxin dynamics with local information on diverse mycotoxin risk factors.

Environmental datasets with high spatial and temporal resolution are increasingly useful for agricultural applications due to improved satellite imagery (e.g., Sentinel-2), more powerful statistical modeling techniques (e.g., machine learning methods), and collaborative efforts to generate agriculturally-relevant metrics (e.g., Famine Early Warning Systems Network (FEWSNET)) [40–43]. Such datasets enable enhanced monitoring of pre-harvest mycotoxin risk factors in rural settings. Combined with georeferenced mycotoxin data, they are vital to developing statistical models that can predict the distribution of mycotoxins across a landscape. For example, in Georgia, USA, weather station data was combined with soil drainage, normalized difference vegetation index (NDVI), and thermal infrared data to model historical aflatoxin contamination in maize [44]. In SSA, NDVI imagery predicted groundnut aflatoxins in Mali, while in Kenya, a combination of NDVI, rainfall, and soil data enabled modeling of maize aflatoxins [45, 46].

In each of these cases, the timing of key indicators of plant stress was also a critical factor, as pre-harvest fungal infection and mycotoxin accumulation are most likely to occur during the flowering and grain fill growth periods [47, 48]. Defining these critical periods helps to improve prediction models of maize aflatoxins in inoculated field trials [36, 49, 50]. Although monitoring the dynamics of pre-harvest environmental factors can be effective and scalable, it cannot account for all drivers of mycotoxin contamination of food, both because mycotoxins can accumulate post-harvest and because many factors are determined at the farm or household scale.

Smallholder maize systems in SSA are complex and diverse in terms of crop genetics, soil conditions, management techniques, processing decisions, and post-harvest handling. Many of these factors influence mycotoxin contamination, and some of them are managed or influenced by actions of farmers and others. Locally sourced information on risk factors and behaviors specific to a farm or community can improve mycotoxin modeling that relies on environmental datasets, and can also help inform better mycotoxin management, including responses to outbreaks. Data on key variables may be ascertained with direct input from the farmers or communities themselves [51]. Indeed, farmer-reported data (variety, fertilizer use, planting, and harvest dates) was key to modeling maize fumonisins in northern Italy [52].

In SSA, information provided by farmers and consumers about production, storage, and processing could also prove useful in modeling mycotoxins. For instance, the application of nitrogen fertilizer reduced maize aflatoxins in Kenyan research trials, suggesting the need to obtain information from farmers about their input use [53]. The post-harvest practice of sorting out moldy, fragmented, or discolored kernels reduces maize fumonisins [22, 54, 55]. In maize processing, dehulling maize grain reduces fumonisin levels, as *F*. *verticillioides* growth is mostly restricted to the outer layers of the grain (the aleurone and pericarp), which are removed during this process [56, 57]. In Tanzania, maize is typically processed as either *dona* flour (whole-grain) or *sembe* flour (dehulled), and in certain regions it is also common to make *kiwerege* flour (soaking dehulled maize in water before milling) [14, 58]. Information about crop management, storage and processing are thus important for assessing mycotoxin risk.

Developing powerful empirical mycotoxin models depends on acquiring a large amount of high-quality mycotoxin data. Mycotoxin assays (e.g. lateral flow devices, enzyme-linked immunosorbent assays (ELISA), etc.) are necessary for any monitoring effort, but their cost and infrastructure requirements often preclude their implementation in low-resource settings [59]. Spectral assays show promise for being an affordable and portable option, particularly in the rural SSA context [60–62]. Although there are tradeoffs in accuracy and a subset of the samples must be tested with validated assays for model training, the ability to use a spectral-based assay to cheaply estimate mycotoxin levels in numerous samples in the field could bolster landscape-wide mycotoxin prediction.

While most prior mycotoxin modeling studies have relied on environmental covariates [46, 63], we hypothesized that a more holistic approach that integrates information on local indicators of mycotoxin risk could be more effective at identifying at-risk areas and guiding mitigation efforts (e.g. breeding resistant varieties, implementing grain sorting practices, improved post-harvest storage methods). This study used a household and grain mill mycotoxin survey in central Tanzania as a platform to test a feasible and multifaceted approach for modeling maize aflatoxins and fumonisins in a smallholder farming system. This approach was based on sampling at local nodes of maize processing, monitoring high-resolution environmental risk factors, surveying farmers’ pre- and post-harvest practices, and using a low-cost spectral device to analyze grain. This effort could provide a framework for mycotoxin monitoring within a low-resource setting, and, ultimately, inform and support mycotoxin mitigation efforts.

## Materials and methods

### Study site

All food surveys occurred in Kongwa District, Tanzania, which has an area of 4,041 square kilometers and a semi-arid agro-pastoral farming system. Among the district’s 309,973 residents, many are smallholder farmers who grow maize as a staple crop in a high temperature and low rainfall (average of 700 mm of rainfall per year) environment [64]. The main crops by cultivated area are maize, millet, sunflower, groundnuts, cassava, and sorghum [64]. This district was also the site of a randomized control trial for testing the effect of aflatoxin exposure on childhood stunting [65].

### 2015 food survey

#### Sampling

The first food survey was conducted from November 10–20, 2015 in 19 villages across Kongwa District. Village leaders led the survey team to households and grain mills where samples of locally processed maize flour samples were obtained. In households where maize flour samples were not available a random sample of whole kernel maize was drawn from pantry stores. Latitude and longitude coordinates of grain mills were acquired in the WGS84 reference coordinate system by using the ODK Collect app, and households were assigned the coordinates of their local grain mill (Get ODK Inc.). Information was collected on the type of maize flour and the source of the maize (homegrown or purchased).

#### Mycotoxin assays

A total of 128 maize samples were collected during this food survey. Total aflatoxins and fumonisins from milled samples were quantified in the field using their respective Neogen Reveal Q+ lateral flow strips. For whole kernel samples, milling and quantification were performed within one week of collection at Nelson Mandela African Institute of Science and Technology (NM-AIST) in Arusha, Tanzania. For aflatoxin and fumonisin extractions, a random 10 g sub-sample of milled maize was placed in 50 ml of 65% ethanol, vortexed for three minutes, and filtered with Whatman #1 filter paper. Then, extracted fumonisins and aflatoxins were assayed according to Neogen kit protocols. Aflatoxins or fumonisins were quantified from their lateral flow strips using the Lab-on-Mobile-Device platform (Mobile Assay Inc.) [66]. Samples that exceeded the Neogen Reveal Q+ limits of detection (2 μg/kg for aflatoxin, and 300 μg/kg for fumonisin) were serially diluted and retested.

### 2019 food survey and farmer questionnaire

#### Sampling and questionnaires

For the 2019 food survey, at least three villages were visited within each ward in Kongwa District. At grain mills, participants were recruited, and interviews were conducted from May 17, 2019 to June 6, 2019. Prior to visiting grain mills, local village leadership was consulted, and they introduced the survey team to the grain mill proprietor. At each grain mill, ODK Collect was used to acquire latitude-longitude coordinates (WGS 84 datum), and then convenience sampling was conducted with the aim of interviewing 3–10 patrons per mill (Get ODK Inc.). Grain mill patrons were informed of the survey’s aims and, if eligible, a brief interview and to a sample of their processed maize meal. The following criteria were applied to identify survey participants: 1) the patron was 18 years of age or older, 2) the maize originated from their local farm, 3) the patron had knowledge on how the maize was grown and how it was handled post-harvest, and 4) the maize was harvested from the current growing season.

Respondents were interviewed about the growing conditions of their maize, their agronomic decisions, and their storage and processing choices, and their responses were recorded in the ODK collect app (S1 Appendix, Get ODK Inc.). Following the interview, the customer’s milled maize was sampled (approximately 300 g), and customers were compensated for their time and maize sample with 2,000 Tanzanian shillings (~US$1). In total, surveyors visited 90 grain mills in 38 villages and conducted 342 surveys, collecting the corresponding number of maize samples.

#### Mycotoxin assays

Milled maize samples were transported to the NM-AIST in Arusha, Tanzania. Samples that were improperly labelled (n = 36) were discarded. Mycotoxin extraction and quantification were conducted on 306 samples according to protocols for total aflatoxins and total fumonisins ELISA kits (Helica Biosystems Inc.).

For each comminuted sample, random subsamples of 9 g and 17 g were measured in separate 50 ml Falcon tubes for aflatoxin and fumonisin extractions, respectively. For aflatoxin extraction, 45 ml of 70% methanol was added, and for fumonisin extraction, 34 ml of 90% methanol was added. Samples were agitated on a shaker for 30 minutes, centrifuged at 10,000 rpm for two minutes, filtered through Whatman #1 filter paper, and 2 ml subsamples of the extracts were stored at 4°C in 2 ml Eppendorf tubes. Following sample extraction, ELISAs for total aflatoxin and total fumonisins were performed according to Helica protocols for each toxin. A BioTek Synergy 2 plate reader with Gen5 software was used to measure the optical density (OD, absorbance at 450 nm) of each well (BioTek Instruments Inc.). A standard curve was fitted to the standards’ OD values, and total aflatoxin or total fumonisin concentrations for each sample were interpolated. If a sample exceeded the ELISA limit of detection (20 μg/kg for aflatoxin and 6 mg/kg for fumonisin), it was diluted and rerun on a new plate. A common sample was assayed on each plate and the between-plate coefficient of variation (CV) threshold was 15%, and duplicate samples within plates were held to a 10% CV threshold. Total aflatoxin and fumonisin values were positively skewed, and a log10-transformation was applied to each prior to inclusion in statistical models (S1 and S2 Tables).

#### NIR analysis

Before mycotoxin extraction, a random subsample of each milled maize sample was scanned three times with a SCiO mini NIR (near-infrared) spectrometer (Consumer Physics). This spectrometer measured reflectance from 740–1070 nm at 1 nm intervals, but because of noise at either end of this spectrum, the range was trimmed to 766–1044 nm. For each sample, the mean reflectance of the three replicate measurements was calculated (S2 Table). To account for light scattering and variability in sensor-sample distance, pre-processing techniques were applied to the mean reflectance values. First, the wavelengths were smoothed using a Savitzky-Golay transformation (differentiation order = 1, polynomial order = 1, window size = 13), and then these transformed wavelengths were normalized using a standard normal variate transformation. A principal components analysis (PCA) was performed on these 279 pre-processed wavelengths.

The NIR data were also used to test whether it could identify different flour types. A random forest classification model (number of trees = 500, variables sampled at each split = 16) was built using all 279 transformed wavelengths as predictors and the three maize types (*dona*, *sembe*, and *kiwerege*) as response variables. The importance of each wavelength was calculated as the percent increase in mean square error (MSE) for permuted out-of-bag data.

### Geospatial datasets acquisition and processing

The elevation of each household or grain mill’s coordinates was acquired from AWS terrain tiles [67]. Environmental datasets were identified based on several criteria including: high spatial resolution (<1 km^2^), previously identified as a pre-harvest risk factor for aflatoxin or fumonisin contamination, and, when applicable, high temporal resolution (≤1 dekad or 10 days). Two datasets that satisfied these criteria had 250 m^2^ spatial resolution: 1) dekadal temporally-smoothed NDVI from FEWSNET, and 2) soil attributes at depth intervals in SoilGrids250m [41, 42]. Based on reported planting and harvest dates in 2019, the maize growing season was defined as spanning December dekad 1 to May dekad 3. For the 2014–2015 and 2018–2019 growing seasons, NDVI values were acquired from the USGS FEWSNET data portal for these 18 dekads.

To ensure that non-farmland signals (e.g. those from inhabited areas, rangeland, bodies of water, brush or forest, etc.) were not included in the analysis, cropland was classified using representative wet season imagery. For each year-dataset combination, non-cropland pixels were masked and excluded from further analyses.

We defined a millshed as the catchment area that was likely to contain farms whose harvests were brought to a given mill. These millsheds were used to capture local environmental variation from high resolution datasets. For the 2015 dataset, NDVI and soil values were extracted from a 5 km radius buffer surrounding each georeferenced mill and averaged (S1 Table). Household samples were assigned the value of their nearest millshed.

In 2019, millsheds were calculated for individual samples by converting reported farm-to-mill walking times into estimated distances from the grain mill. To find estimated distances the reported time was divided by a 3 km/hour walking speed. Further restrictions were a 0.5 km minimum and 10 km maximum on walking distances. Any unreported distances were assigned the mean walking distance. These distances were used to create a distance buffer radius from the grain mill for extracting and averaging soil and NDVI values for each sample (S2 Table).

A PCA was performed on each year-dataset combination; in total, four separate PCAs were run on the extracted and buffer-averaged values from the 2015 and 2019 NDVI and soil datasets. The number of PCs in models was determined by including those that explained up to 90% of cumulative variation for a given dataset.

### 2015 mycotoxin models

To assess the influence of survey and environmental factors on maize mycotoxins from the initial 2015 survey, multiple linear regression models were constructed using these potential mycotoxin risk factors as covariates. The response for each model was log10-transformed aflatoxin or log10-transformed fumonisin values, and the covariates included flour type, source (homegrown vs. purchased), household elevation, the first five principal components of all eleven of soilGrids250m attributes, and the first three principal components of the entire growing season’s dekadal NDVI values. Then, to test whether environmental factors were more effective at modeling mycotoxins in homegrown maize compared to purchased maize, the same models (with the exception of “source”) were constructed using only homegrown or only purchased samples.

### 2019 mycotoxin models

One-way analysis of variance (ANOVA) was used to test the effect of maize flour type on transformed aflatoxin or fumonisin levels within each year. If significant, post-hoc pairwise t-tests between each group were performed and the Holms method was used to adjust for multiple comparisons.

Mixed linear models were used to model aflatoxins and fumonisins. First, a mixed linear model was built using the first five NIR PCs, the first three NDVI PCs, the first five soil PCs as fixed effects and mill village as a random effect. Before constructing linear mixed models, the mean at each of the 88 grain mills was calculated for all attributes of the 306 samples, and these values were used for the response and predictor variables.

### Software and data analysis

Data processing and statistical analysis were conducted in R version 4.3.1 [68]. For NIR data, the *prospectR* package was used to pre-process NIR wavelengths [69]. For environmental data processing, the *raster* package was used [70]. For linear mixed models, the *lme4* package was used to construct the models, the *lmerTest* package was used to identify significant covariates, and the *MuMin* package was used to calculate conditional and marginal coefficients of determination [71–73]. Random forest modeling and variable importance calculations were performed using the *randomForest* package in R [74]. Elevation data for each location were acquired using the *elevatr* package in R [67]. Data were visualized using the *ggplot2* and *ggpubr* R packages [75, 76].

### Ethical considerations

Cornell University’s Institutional Review Board provided the ethical approval for farmer questionnaires (Protocol ID# 1809008284). As documented in the approval’s standard operating procedure for this study, individual farmer interviews were conducted in Swahili. Research team members read a script to potential participants explaining the purpose of the study and the criteria for participant inclusion, and, if they qualified, their consent for a sample and a brief interview was politely solicited. Oral informed consent was documented by the lead member of the research team on the data form and witnessed by the second member of the team. No identifying information was recorded, and data were analyzed anonymously. Administrative permission to visit local grain mills was received from the Kongwa District Executive Director.

## Results

### Kongwa District survey insights

Many preliminary indicators suggested that the residents of Kongwa District would be at high risk of mycotoxin exposure. In 2019, we characterized the local context with a farmer questionnaire, both to better understand mycotoxin risk factors and to better envisage management options. In 2015, the food survey accounted only for whether the maize was homegrown (59.8%) or purchased (40.2%) and the type of maize flour (*dona* = 37.6%, *sembe* = 53.0%, *kiwerege* = 9.4%). The 2019 food survey only sampled homegrown maize and used a more extensive questionnaire to gain insights into pre-harvest factors, agronomic decisions, post-harvest handling, and processing decisions (Table 1). Kongwa residents produce maize for either subsistence use and/or to sell at the market. Self-provisioned maize was commonly processed at grain mills that were located within walking distance of their farms (mean = 77.0 minutes and median = 60.0 minutes).

**Table 1:** Summarized responses from the 2019 questionnaire of grain mill customers.

2019 Survey Question (# respondents)	2019 Survey Response (% of respondents)				
Time to walk from farm to mill? (N = 293)	0–1 hour (69.6%)	1–2 hours (22.2%)	2–3 hours (4.1%)	3+ hours (4.1%)	
Seed source? (N = 318)	Saved from previous year’s harvest (90.9%)	Purchased from neighbor or store (9.1%)			
Intercropped? (N = 330)	Yes, maize was intercropped with another crop (77.9%)	No (22.1%)			
Number of intercropped crops with maize? (N = 249)	1 (55.8%)	2 (32.9%)	3 (8.4%)	4–6 (1.2%)	
Intercrop crop type? (N = 249)	Oil Crop (65.4%)	Legume (52.9%)	Grain (32.3%)	Fruit/Vegetable (5.4%)	Tuber (3.9%)
Planting month? (N = 328)	December 2018 (58.2%)	January 2019 (36.3%)	November 2018 (3.0%)	February 2019 (1.8%)	October 2018 (0.061%)
Harvest month? (N = 329)	May 2019 (81.2%)	June 2019 (9.4%)	April 2019 (7.9%)	March 2019 (1.9%)	
Fertilizer use in 2018–2019 season? (N = 316)	None (91.8%)	Animal manure (7.6%)	Chemical fertilizer (0.63%)		
Drought stress during maize growing season? (N = 329)	Yes (97.8%)	No (2.1%)			
Drought stress period? (N = 321)	Middle (61.4%)	Middle/End (23.1%)	End (7.5%)	Beginning/Middle (4.0%)	Other (4.0%)
Relative pest pressure? (N = 328)	Normal (41.5%)	Less severe than normal (32.9%)	More severe than normal (25.6%)		
At harvest, was the maize fully matured and dried in field? (N = 329)	Yes, matured and dried (57.8%)	No, not matured. Yes, dried. (18.2%)	Yes, matured. No, not dried. (17.6%)	Not matured and not dried. (6.4%)	
Matured maize was rained on prior to harvest? (N = 247)	Yes (93.1%)	No (6.9%)			
When was the maize shelled in relation to drying? (N = 239)	Before drying (58.4%)	After drying (24.6%)	Partially dried, shelled, dried completely (17.0%)		
Maize storage? (N = 289)	Plastic bags (68.7%)	None, brought to mill directly from harvest (18.5%)	Metal or hard plastic container (7.3%)	Other (5.5%)	
Cleaning and sorting? (N = 287)	Winnowed and hand-sorted (80.8%)	Winnowed only (11.1%)	None (5.2%)	Hand-sorted only (2.8%)	
Processing and milling? (N = 300)	*Sembe*: Dehulled and milled (64.0%)	*Dona*: Whole grain milled (30.7%)	*Kiwerege*: Dehulled, soaked in water, milled (5.3%)		

Maize was planted as early as the beginning of October 2018, but most farmers planted in December 2018 or January 2019. Farmers explained that their planting decisions were guided by local rainfall patterns and their individual risk tolerance. Planting immediately after the initial rains in early December was described as a higher risk decision, while those with moderate risk tolerance planted in late December, and the most risk averse waited until January to plant after rains had definitively established. Season length (harvest dekad-planting dekad) had a median of 14 dekads (~140 days), but maize was in the field for as little as 8 dekads and as long 22 dekads. These season lengths can be explained in part by farmers allowing their maize to dry in the field and harvesting portions of their field as needed.

Farmers used very few inputs. Most seed had been saved from the previous year, fertilizer use was rare, and there were no reports of irrigation. In terms of pre-harvest conditions, nearly all farmers reported that their maize experienced drought-like conditions, especially in the middle of the season (February-March). Though there is commonly a short break in rains in January or February, the intensity and duration were described as more severe than normal in the 2018–2019 growing season. Most farmers allowed their maize to mature and dry in the field before harvesting, and they reported that this matured and dried maize was rained on. Farmers grew their maize in diverse cropping systems with two to six other crops intercropped with their maize. Leguminous and oil crops were the most common classes of crops, with the cash crop sunflower being the most common choice for intercropping.

Following harvesting and drying, maize was typically stored in polypropylene bags, though some maize was brought directly to the mill for processing. The vast majority of those interviewed winnowed and hand-sorted their maize prior to milling, but we observed that the intensity of hand-sorting varied (e.g. from quickly removing a few rocks and cobs to painstakingly removing all abnormal kernels).

Respondents processed their maize using different methods: 1) milling whole grain (*dona*), 2) dehulling and milling (*sembe*), and 3) dehulling, soaking in water for at least one night, and then milling (*kiwerege*). These processing choices were influenced by a variety of factors including food security, labor required, food quality, and personal preference. For instance, some would opt to go to certain mills based on their preference for how much bran and germ would be removed in the dehulling process.

### Kongwa District environmental insights

**Fig 1:**
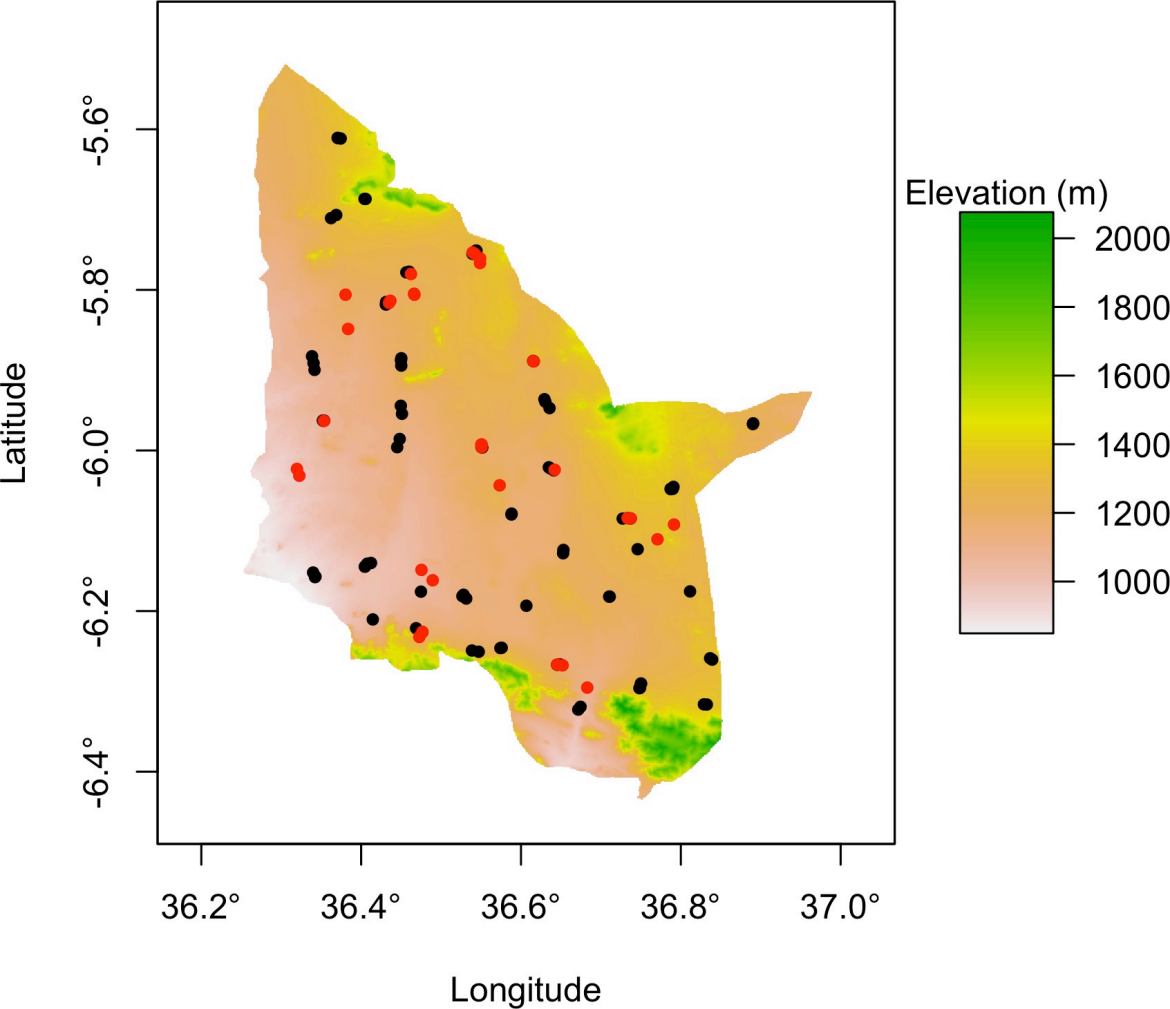
Sampling sites were distributed widely across Kongwa District. Elevation (meters above sea level) map of Kongwa District with 32 millsheds sampled in 2015 (red dots) and 90 millsheds sampled in 2019 (black dots).

To further contextualize mycotoxin risk factors in Kongwa District, we characterized the environmental conditions under which the maize was likely grown. Sampling occurred across the entirety of Kongwa District where the elevation of surveyed grain mills had a mean and median of 1,214 and 1,224 masl and a minimum and maximum of 904 and 1,492 masl (Fig 1).

**Fig 2:**
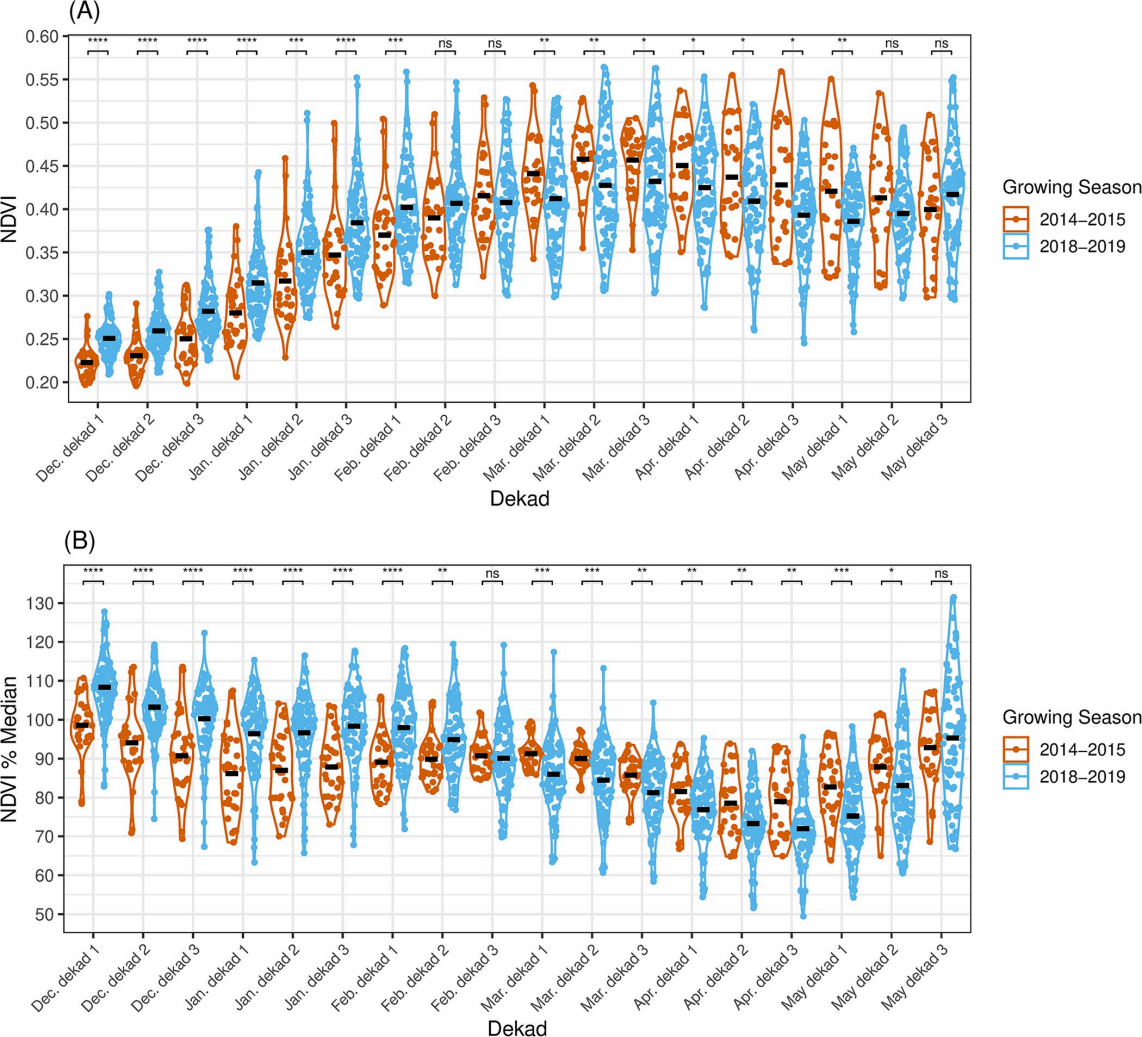
Kongwa District vegetation dynamics vary across the 2014–15 and 2018–2019 growing seasons. (A) Millshed mean NDVI dekadal values across the 2014–2015 (n = 32) and 2018–2019 (n = 90) maize growing seasons in Kongwa District (black lines = dekad mean). At each growing season dekad, a two-sample t-test was run to test for differences between NDVI values in 2015 and 2019, and p-values were adjusted for multiple comparisons with the Holm method (ns p>0.05, * p<0.05, ** p<0.01, *** p<0.001, **** <0.0001). (B) Millshed mean NDVI values as a percentage of the median NDVI from 2003–2017 (black lines = dekad mean, dashed line = equal to median of 2003–2017). At each dekad, a two-sample t-test was run to test for differences between NDVI percentage median values in 2015 and 2019, and p-values were adjusted with the Holm method (ns p>0.05, * p<0.05, ** p<0.01, *** p<0.001, **** <0.0001).

NDVI values extracted from millshed buffers illustrated the dynamics of vegetation growth across the maize growing seasons. For instance, maximum mean millshed NDVI values were observed during the final two dekads of March, which roughly indicates the flowering time of maize, as this demarcates a transition between maize’s vegetative and reproductive growth phases (Fig 2). Comparing the two seasons, NDVI values diverged halfway through the growing season; 2015 NDVI values were higher than those in 2019 from the end of February to mid-May. The 2019 NDVI values were consistent with farmers’ characterization of the middle of the growing season as having little to no rainfall; following this period (February to March 2018), there was a flattening of NDVI, which suggested that plant health was negatively affected by the reported lack of rain. Relative to the norm (median NDVI between 2003–2017), both seasons had depressed NDVI from the middle to end of the growing season (March-May), and this plant health deficit was particularly pronounced in the 2018–2019 growing season. This indicated that maize crops were stressed during the maize flowering and grain fill periods.

**Fig 3:**
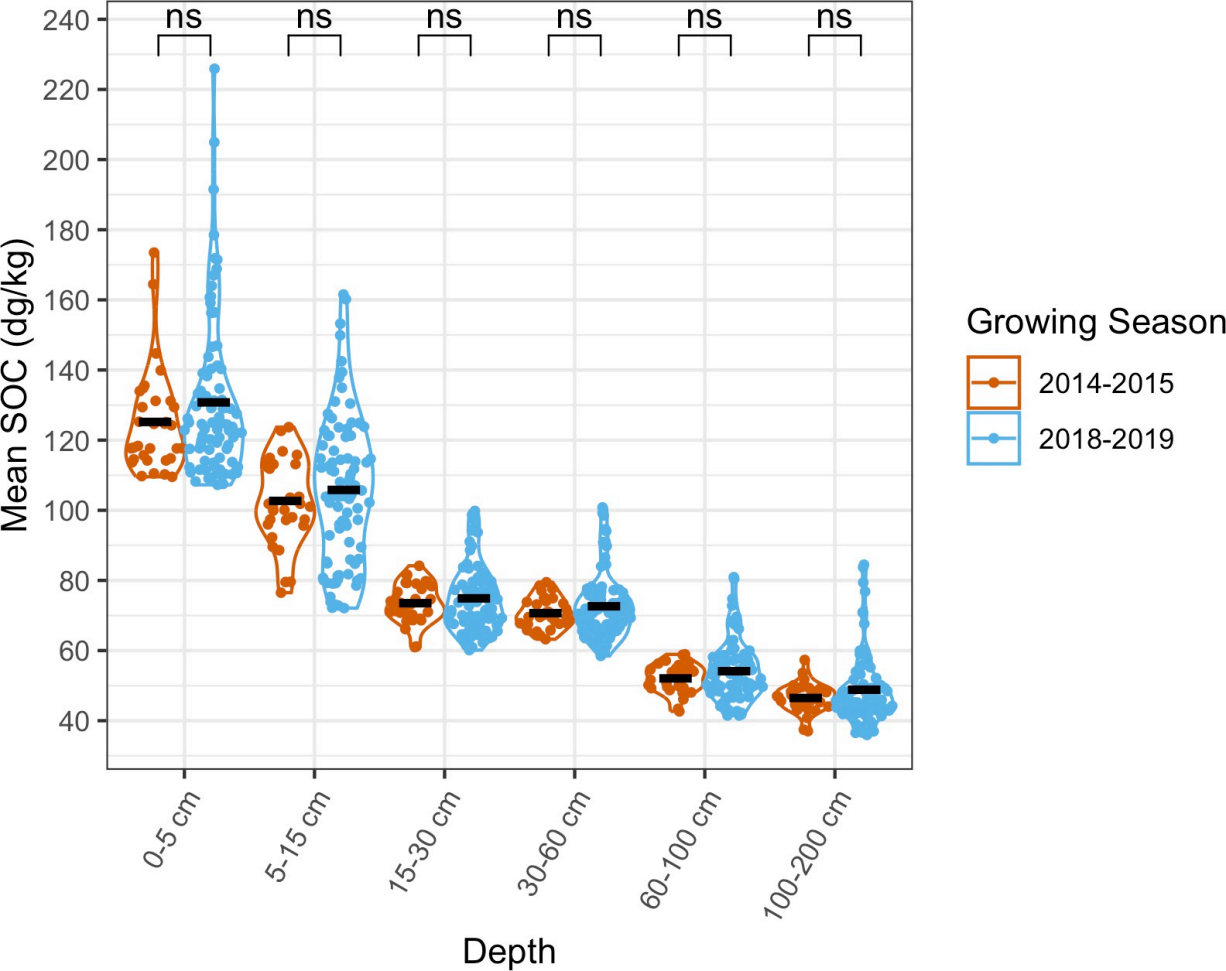
Soil organic carbon values of millsheds did not differ between the two survey years. Millshed mean soil organic carbon (SOC) in the fine earth fraction across depths for the 2014–2015 and 2018–2019 maize growing seasons (black lines = depth mean). At each soil depth, a two-sample t-test was performed to test for differences in SOC means in 2015 and 2019, and p-values were adjusted with the Holm method (ns p>0.05).

Soil plays a vital role in supporting plant health and can serve as a buffer against potential stressors. Whereas a pre-harvest factor like NDVI varies within seasons and across years, available soil data were static relative to time. Assessment of a soil factor that has been negatively associated with mycotoxin contamination, soil organic carbon (SOC), showed that millsheds’ values did not differ significantly across years (Fig 3).

### Kongwa District NIR insights

**Fig 4:**
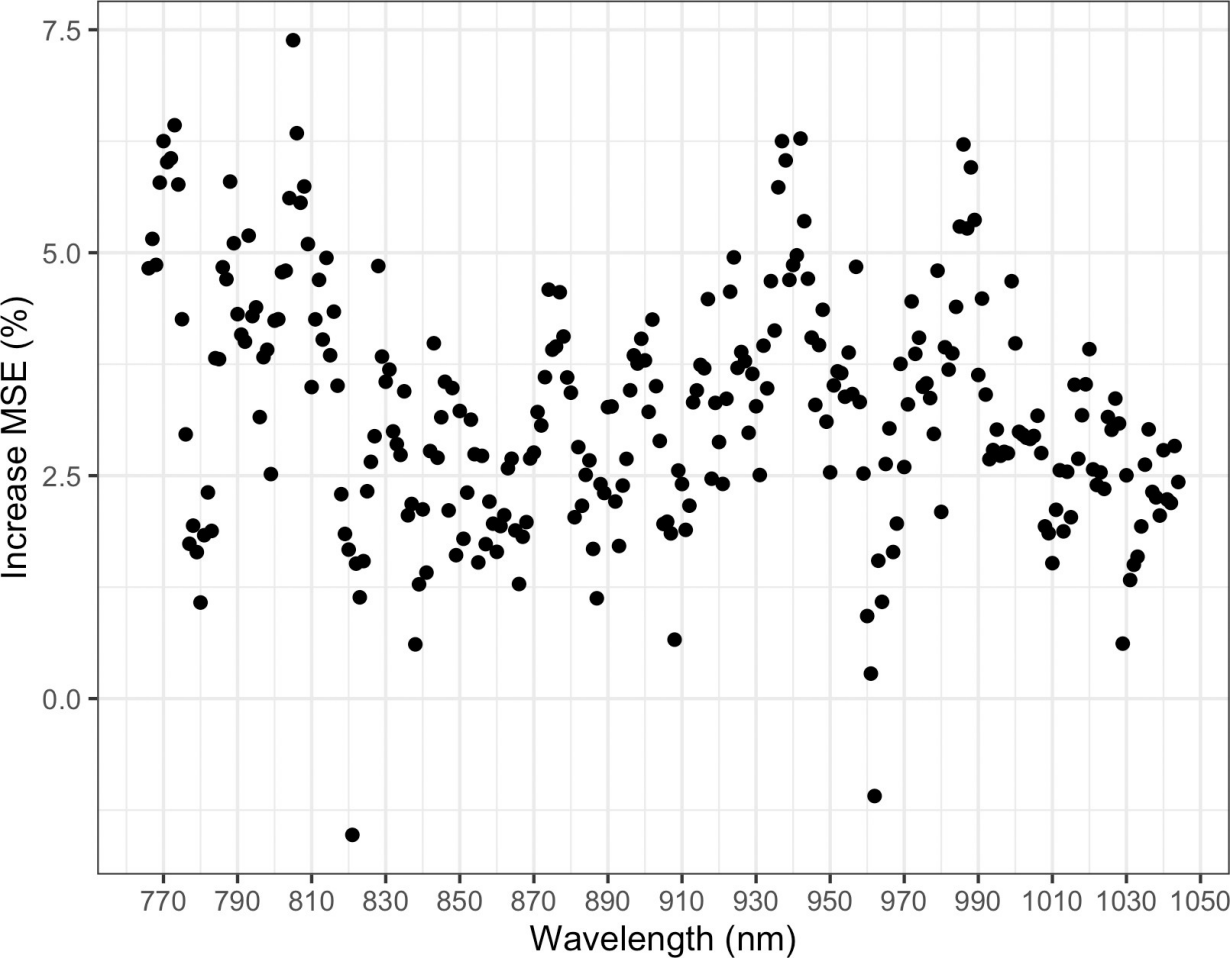
Specific NIR wavelengths disproportionately influence flour type classification. Percent increase in mean square error (MSE) for all near-infrared wavelengths in a random forest classification model for maize flour type.

Using NIR measurements of maize flour samples, random forest classification was able to classify flour type with an out-of-bag error rate of 10.3%. For the different flour types, error rates were 12.0% for *dona*, 7.8% for *sembe*, and 31.3% for *kiwerege*, though low accuracy for *kiwerege* identification was likely influenced by its small number of samples (n = 16). Measuring percent increase MSE showed that 773 and 805 nm were the most important NIR wavelengths for flour type identification (Fig 4).

### Mycotoxins and surveyed risk factors

**Fig 5:**
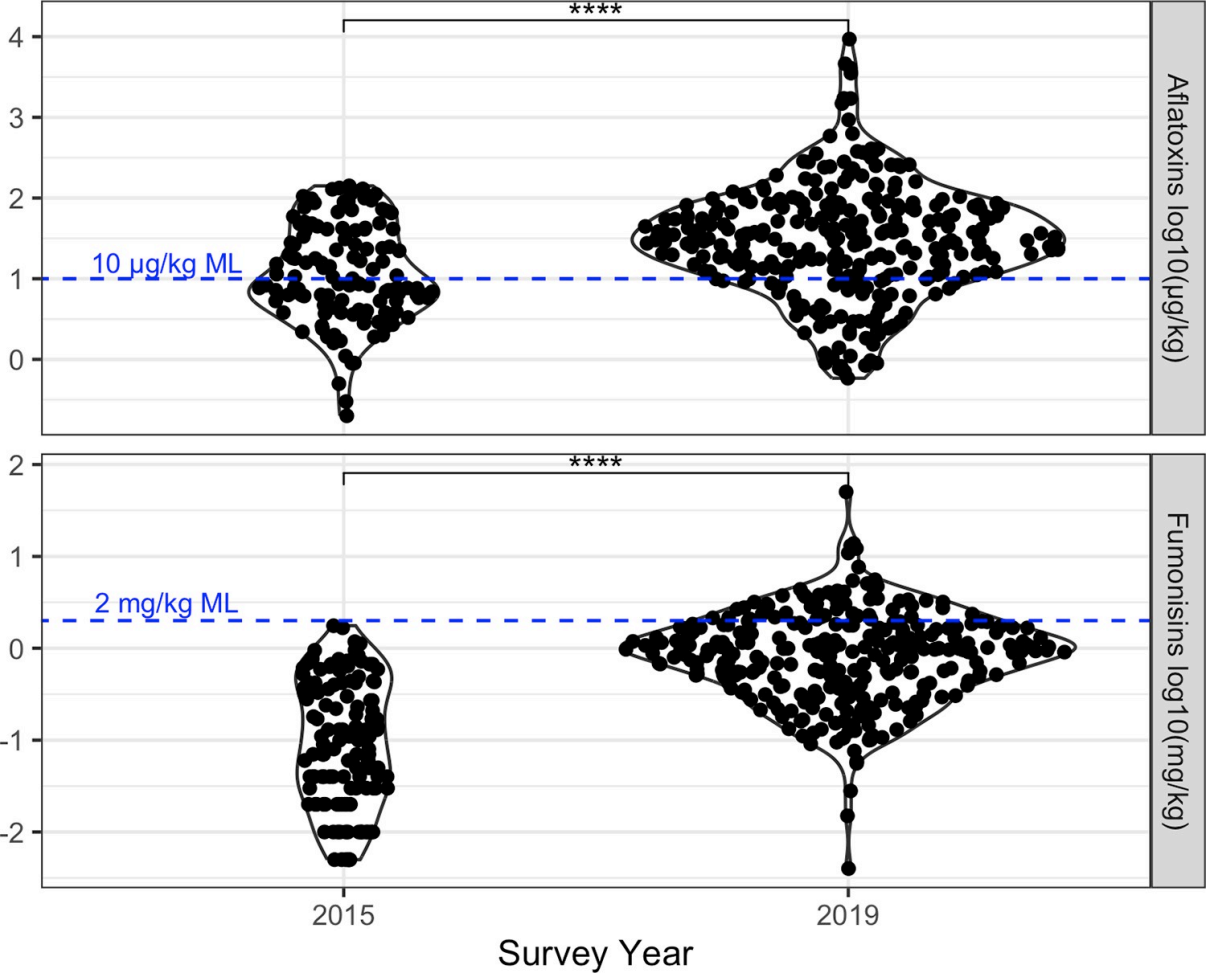
Aflatoxins and fumonsins were elevated in 2019 compared to 2015. Log10-transformed total aflatoxin (top) and total fumonisin (bottom) concentrations in all milled maize samples from the 2015 and 2019 Kongwa District surveys. Blue dashed lines indicate the maximum limits (ML) for total aflatoxins (10 ug/kg) and fumonisins (2 mg/kg) in maize flour. There were significantly higher concentrations of aflatoxins and fumonisins in 2019 than in 2015 (two-sample t-tests, **** p<0.0001).

In 2015, milled maize samples collected from grain mills and household stores had a geometric mean (95% CI) of 11.6 μg/kg (9.1–14.7 μg/kg) for maize aflatoxins, and 0.094 mg/kg (0.071–0.12 mg/kg) for maize fumonisins. Maize mycotoxin contamination was more severe in 2019 than in 2015, with a geometric mean (95% CI) of 29.9 μg/kg (25.0–35.7 μg/kg) for total aflatoxins and 0.80 mg/kg (0.70–0.99 mg/kg) for total fumonisins (Fig 5). In relation to the East African Community’s standards for maize flour, a substantial percentage of 2015 (47.7%) and 2019 (78.4%) samples exceeded the 10 ppb (μg/kg) maximum level for total aflatoxins. For total fumonisins, the 2 ppm (mg/kg) maximum limit was not exceeded by any samples in 2015, but it was exceeded by 18.6% of 2019 samples [77].

**Fig 6:**
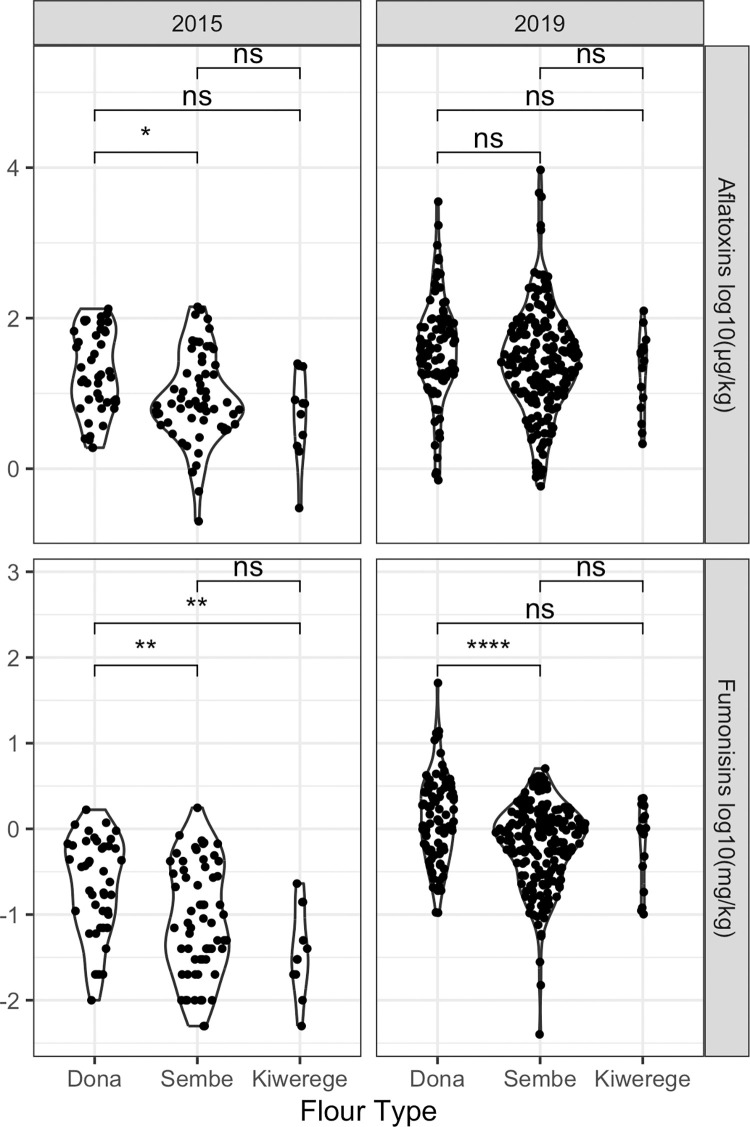
Maize aflatoxin and fumonisin levels varied according to flour type. Divided by flour type, the log10-transformed total aflatoxin (top) and total fumonisin (bottom) concentrations in milled maize from the 2015 and 2019 Kongwa District surveys. Mycotoxin concentrations varied significantly across flour types (1-way ANOVA F<0.05) for 2015 aflatoxins, 2015 fumonisins, and 2019 fumonisins. *Sembe* (dehulled) flour had lower aflatoxins (2015) and fumonisins (2015 and 2019) than *dona* (whole grain), and *kiwerege* (dehulled and soaked) had lower fumonisins than *dona* in 2015 (two-sample t-test with Holm multiple testing adjustment, ns p>0.05, * p<0.05, ** p<0.01,*** p<0.001,**** p<0.0001,).

The questionnaires were intended to interrogate various potential risk factors of mycotoxin contamination, and some survey responses were significantly associated with aflatoxin or fumonisin contamination. Flour type was associated with fumonisins in both 2015 and 2019; dehulled flours (*sembe* and *kiwerege*) had lower concentrations than whole grain flours (*dona*). This same reduction from *dona* to *sembe* was also observed for aflatoxin, but only among 2015 samples (Fig 6).

### Modeling maize aflatoxins and fumonisins

Linear regression models from the 2015 survey offered key insights into the drivers of maize aflatoxin and fumonisin contamination. In modeling all samples, certain effects were shared between aflatoxin and fumonisin models; purchased maize increased aflatoxins and fumonisins, while *kiwerege* flour had a negative effect on both, and *sembe* flour was negatively associated only with fumonisins (Tables 2 and 3).

**Table 2:** Summary statistics of linear models for aflatoxins from the 2015 survey.

	2015 All Maize Aflatoxins log10(ug/kg)			2015 Homegrown Maize Aflatoxins log10(ug/kg)			2015 Purchased Maize Aflatoxins log10(ug/kg)		
Predictors	Estimates	CI	p	Estimates	CI	p	Estimates	CI	p
(Intercept)	-1.47	-5.12 – 2.18	0.427	-4.54	-10.25 – 1.17	0.117	2.35	-3.81 – 8.51	0.444
Flour type [dehulled]	-0.03	-0.28 – 0.21	0.786	0.03	-0.27 – 0.33	0.847	-0.16	-0.56 – 0.25	0.439
Flour type [dehulled-soaked]	-0.47*	-0.83 – -0.10	**0.012**	-0.53*	-1.04 – -0.03	**0.038**	-0.39	-1.00 – 0.22	0.200
Source [purchased]	0.25*	0.04 – 0.47	**0.021**						
Household elevation (m)	0.00	-0.00 – 0.01	0.181	0.00	-0.00 – 0.01	0.052	-0.00	-0.01 – 0.00	0.730
2015 Soil PC1	-0.04	-0.10 – 0.02	0.170	-0.13**	-0.21 – -0.04	**0.004**	0.05	-0.05 – 0.15	0.310
2015 Soil PC2	0.09***	0.04 – 0.13	**<0.001**	0.12***	0.06 – 0.17	**<0.001**	0.06	-0.03 – 0.14	0.182
2015 Soil PC3	-0.03	-0.10 – 0.04	0.450	-0.02	-0.11 – 0.08	0.737	-0.01	-0.13 – 0.10	0.834
2015 Soil PC4	-0.02	-0.08 – 0.03	0.426	-0.05	-0.11 – 0.01	0.129	0.02	-0.09 – 0.14	0.656
2015 Soil PC5	-0.05	-0.11 – 0.02	0.143	-0.06	-0.14 – 0.03	0.177	-0.03	-0.16 – 0.11	0.707
2015 NDVI PC1	-0.03	-0.10 – 0.05	0.450	-0.07	-0.17 – 0.03	0.165	0.03	-0.09 – 0.15	0.660
2015 NDVI PC2	-0.03	-0.09 – 0.04	0.421	0.00	-0.13 – 0.14	0.960	-0.07	-0.18 – 0.03	0.176
2015 NDVI PC3	-0.01	-0.12 – 0.11	0.914	-0.08	-0.22 – 0.06	0.241	0.11	-0.10 – 0.33	0.288
Observations	116			66			50		
R^2^	0.352			0.471			0.360		
Adjusted R^2^	0.276			0.364			0.175		
Deviance	26.875			11813			9.640		

All three models were significant (F-test, p<0.05).

*p<0.05

**p<0.01

***p<0.001

**Table 3:** Summary statistics of linear models for maize fumonisins from the 2015 survey.

	2015 All Maize Fumonisins log10(mg/kg)			2015 Homegrown Maize Fumonisins log10(mg/kg)		
*Predictors*	*Estimates*	*CI*	*p*	*Estimates*	*CI*	*p*
(Intercept)	2.06	-2.10 – 6.22	0.329	4.94	-2.85 – 12.73	0.209
Flour type [dehulled]	-0.37**	-0.66 – -0.09	**0.010**	-0.33	-0.74 – 0.08	0.112
Flour type [dehulled-soaked]	-0.69**	-1.10 – -0.28	**0.001**	-0.66	-1.34 – 0.03	0.059
Source [purchased]	0.26*	0.01 – 0.50	**0.038**			
Household elevation (m)	-0.00	-0.01 – 0.00	0.169	-0.00	-0.01 – 0.00	0.142
2015 Soil PC1	0.05	-0.02 – 0.12	0.175	0.05	-0.06 – 0.17	0.360
2015 Soil PC2	-0.05	-0.10 – 0.00	0.066	-0.05	-0.13 – 0.03	0.200
2015 Soil PC3	0.01	-0.07 – 0.09	0.851	0.01	-0.12 – 0.13	0.932
2015 Soil PC4	-0.01	-0.08 – 0.05	0.728	-0.01	-0.09 – 0.08	0.904
2015 Soil PC5	0.01	-0.06 – 0.09	0.782	0.00	-0.11 – 0.12	0.981
2015 NDVI PC1	-0.00	-0.09 – 0.08	0.930	-0.04	-0.18 – 0.10	0.556
2015 NDVI PC2	-0.01	-0.08 – 0.07	0.810	-0.08	-0.26 – 0.11	0.402
2015 NDVI PC3	-0.07	-0.20 – 0.06	0.305	-0.16	-0.35 – 0.02	0.081
Observations	116			66		
R^2^	0.289			0.311		
Adjusted R^2^	0.206			0.170		
Deviance	34.906			21.997		

The purchased maize fumonisins model was not (F-test, p>0.05), so results were not included in this table.

Only the all samples and homegrown maize fumonisin models were significant (F-test, p<0.05).

*p<0.05

**p<0.01

***p<0.001

The aflatoxin model with only homegrown samples compared favorably to the aflatoxin model of only purchased samples, which was not significant. Also, the homegrown-only aflatoxin model explained more variation and identified more significant covariates than the full model: dehulled-soaked (*kiwerege*) flour, Soil PC1, and Soil PC2 (Table 2). This suggested that focusing only on homegrown maize could better model aflatoxins and identify more environmental risk factors.

When fumonisin data was divided into purchased and homegrown subsets, the homegrown model was significant (p-value = 0.027, but had no significant covariates, while the purchased model was not significant (p = 0.24) (Table 3). Together, the 2015 linear regression models showed that both survey and environmental factors explained variation in maize aflatoxins and fumonisins.

Nearly three times as many samples were used in the 2019 survey, and they were collected only at grain mills. Due to study design changes (e.g. a larger dataset with increased sampling per grain mill), the 2019 survey was better suited to the nested structure of a linear mixed model in which village was used as a random effect. The 2019 linear mixed model explained over a third of variation in aflatoxins, and it identified NIR PC1 and NDVI PC3 as significant covariates (Table 4). For maize fumonisins, a similar proportion of variation was explained using the same predictors, however, different significant covariates were identified (NIR PC3, NDVI PC1, NDVI PC2) (Table 4). In both models, a small amount of variation was explained by the random effects (mill village), suggesting that some local variation contributing to mycotoxin contamination was not captured by NIR, NDVI, or soil factors.

**Table 4:** Summary statistics of mixed linear models for maize aflatoxins and fumonisins from the 2019 survey.

	2019 Maize Aflatoxins log10(ug/kg)			2019 Maize Fumonisins log10(mg/kg)		
*Predictors*	*Estimates*	*CI*	*p*	*Estimates*	*CI*	*p*
(Intercept)	1.52***	1.40 – 1.64	**<0.001**	-0.09*	-0.16 – -0.02	**0.010**
NIR PC1	0.02 **	0.01 – 0.04	**0.003**	0.00	-0.00 – 0.01	0.321
NIR PC2	0.01	-0.00 – 0.03	0.143	-0.01	-0.02 – 0.00	0.064
NIR PC3	-0.00	-0.03 – 0.02	0.774	0.02*	0.00 – 0.03	**0.036**
NIR PC4	0.03	-0.02 – 0.07	0.224	-0.00	-0.03 – 0.02	0.722
NIR PC5	-0.04	-0.09 – 0.02	0.212	-0.02	-0.05 – 0.01	0.169
2019 NDVI PC1	-0.01	-0.06 – 0.05	0.799	0.04*	0.01 – 0.07	**0.013**
2019 NDVI PC2	-0.03	-0.11 – 0.05	0.464	0.06*	0.01 – 0.10	**0.012**
2019 NDVI PC3	-0.19**	-0.31 – -0.06	**0.004**	0.06	-0.01 – 0.14	0.090
2019 Soil PC1	0.03	-0.00 – 0.06	0.071	-0.01	-0.03 – 0.01	0.295
2019 Soil PC2	-0.01	-0.06 – 0.03	0.569	-0.02	-0.05 – 0.00	0.076
2019 Soil PC3	0.04	-0.02 – 0.09	0.178	0.01	-0.02 – 0.04	0.493
2019 Soil PC4	-0.04	-0.09 – 0.01	0.115	0.02	-0.01 – 0.05	0.244
2019 Soil PC5	0.01	-0.05 – 0.06	0.859	-0.02	-0.06 – 0.01	0.184
**Random Effects**						
σ^2^	0.26			0.08		
	0.02 (mill village)			0.01 (mill village)		
ICC	0.07			0.11		
N	38 (mill village)			38 (mill village)		
Observations	88			88		
Marginal R^2^	0.350			0.324		
Conditional R^2^	0.394			0.400		

*p<0.05

**p<0.01

***p<0.001

As in the 2015 survey, the 2019 models showed the influence of pre-harvest environmental factors on maize aflatoxin and fumonisin levels. NDVI covariates were more influential in 2019 models, perhaps because there was more variation in NDVI during this growing season. Furthermore, the 2019 models demonstrated the utility of including proximate sensing predictors, in the form of NIR reflectance measures, in complementing pre-harvest environmental factors.

## Discussion

Understanding where, when, and to what extent mycotoxin contamination affects local communities is a necessary first step toward implementing scalable mitigation efforts. Over the course of two food surveys, we documented significant maize aflatoxin and fumonisin contamination within a Tanzania smallholder farming system. We sought to emulate the structure of a potential mycotoxin monitoring system by modeling these mycotoxins as a function of survey data, spectral grain analysis (NIR), and environmental factors.

Prior efforts to model mycotoxins with environmental data in SSA have sampled across agroecological zones and wide ranges of pre-harvest risk factors like soil quality, NDVI, rainfall, and temperature [46, 63, 78]. By contrast, this study entailed denser sampling within a semi-arid smallholder system. Even within a relatively narrow range of environmental variation, soil and NDVI factors explained variation in maize aflatoxins and fumonisins. Further, models for aflatoxins and fumonisins identified different significant environmental factors, which underscores the theory that although they frequently co-occur, pre-harvest aflatoxin and fumonisin contamination is differentially affected by the environment [21, 78]. In the future, the use of environmental data could be bolstered by higher temporal resolution datasets, mycotoxin-specific risk factors (e.g. drought stress index), and more precise knowledge of key events like planting date, flowering date, and harvest date.

We documented higher levels of maize aflatoxin and fumonisin contamination in the 2019 survey, and pre-harvest abiotic stress likely influenced this outcome. The plant health of millsheds in the 2018–2019 growing season, as measured by dekadal NDVI, was reduced compared to both the 2014–2015 season and to the historical norm. These reductions were especially prominent during key flowering, grain fill, and maturity periods. This reinforces the evidence that maize under abiotic stress during these critical periods is more susceptible to aflatoxin and fumonisin contamination [38, 39]. In 2019, farmer questionnaires also provided insights on environmental drivers of mycotoxin accumulation, as the majority reported that their matured maize was rained upon. Late season rains have been implicated as a pre-harvest risk factor for aflatoxins, as they promote *A*. *flavus* growth on already infected ears [38].

Our findings regarding maize processing in Kongwa District highlighted the interconnected tensions among food safety, food security, domestic labor, and cultural preferences. In Tanzania, dehulled maize flours (*sembe* or *kiwerege*) are often preferred as they produce whiter and softer *ugali* compared to whole grain flour (*dona*) [58]. Dehulled flours also take more time and labor, especially in the production of the highly valued *kiwerege*, which requires soaking dehulled maize in water for 1–6 days prior to milling [58]. From food security and nutritional perspectives, the dehulling process removes a significant fraction of edible maize (5–25% for *sembe* and 30–40% for *kiwerege*), and these dehulled flours are more carbohydrate-dense since they lack the nutrient-rich germ and bran [14]. Our findings indicated a preference for dehulled maize flours in Kongwa District, and confirmed the mycotoxin-reduction effect of dehulling, particularly for fumonisins [56, 58]. Processing techniques like dehulling should be accounted for, not only when monitoring maize mycotoxins, but also when considering them as part of mitigation efforts, as there are accompanying implications on food quality, food security, and local preferences.

Cost-effective and portable spectral devices, like the SCiO used in this study, have been tested for a variety of agricultural applications such as cultivar identification, cassava starch phenotyping, and quantifying milk nutrients [79–81]. We further demonstrated the utility of a relatively low-cost NIR spectrometer by using spectral information to both classify different maize flour types and model a portion of variation in maize aflatoxins and fumonisins. NIR signatures capture a wealth of information on the biochemical composition of maize grain, and they could help to model mycotoxins by indirectly accounting for associated risk factors (flour type, variety, soil quality). Future work could substantially improve on these efforts with improved spectrometers and larger sample sizes, and improved spectral detection of mycotoxins should be prioritized to expand mycotoxin monitoring, ideally validated by gold-standard quantification methods.

Homegrown maize has been cited as a prominent source of aflatoxins during notable aflatoxicosis outbreaks in eastern Kenya and in central Tanzania, close to where this survey occurred [11, 82, 83]. Although homegrown maize from the eastern Kenya hotspot had higher in aflatoxins than purchased maize, this study and others have documented the opposite, which suggests that this phenomenon is context-dependent [53, 83]. This is supported by observations in Kenya that maize farmers are more likely to sell lower quality grain with higher aflatoxin concentrations to markets and retain higher quality grain for home consumption [84]. Ultimately, the level of toxicity is likely to depend on a complex interaction of factors such as the source of the purchased maize, the toxicity of local fungal strains, the season during which samples are collected, and the storage quality and duration, among others [82, 85–87].

Ideally, mycotoxin monitoring could rapidly and efficiently identify hotspots and inform intervention efforts on how to respond in the most effective and appropriate manner; doing so would entail integrating community-level insights with centralized resources and capabilities in an appropriately flexible fashion [88]. Recent efforts to monitor other agricultural challenges in low-resource areas have deployed mobile apps that enable farmers to supply information and receive customized advice. PlantVillage’s Nuru app uses farmer-supplied images to identify the pests and diseases affecting their crops (e.g., viral diseases of cassava, fall armyworm, locusts), and the same images help improve prediction models based on remote-sensing environmental data [89]. Other lessons can be learned from plant disease surveillance efforts, such as MARPLE, which uses field-based genomics to rapidly identify fungal pathogen strains [90]. Mycotoxin monitoring schemes are difficult to implement due to the cost and infrastructure requirements for mycotoxin detection, but the frameworks and principles applied in Nuru and MARPLE could substantially inform these efforts.

This study aimed to serve as an example for how future monitoring efforts can better understand mycotoxin risk in smallholder agricultural contexts. We validated the importance of key elements of such a mycotoxin monitoring network: sampling at local nodes of maize processing, contextualizing the system with local feedback, capturing high resolution environmental variation, and using portable low-cost spectral sensors of grain quality. This work underscored the importance of using multiple indicators of mycotoxin risk in resource-limited and decentralized food systems. Scaling mycotoxin monitoring within smallholder settings is critical to predicting mycotoxin hotspots and ensuring a rapid response to a potential mycotoxicosis outbreak. It can also help to identify persistent factors that drive mycotoxin exposure and use them as a platform for sustainable mitigation efforts. This study demonstrated that integrating information on diverse risk factors bolstered mycotoxin modeling and improved understanding of mycotoxin dynamics.

## Supporting information

S1 Appendix 2019 grain mill survey questions in English and Swahili. (DOCX)

S1 Table Compiled table of maize samples’ associated locations, mycotoxin concentrations, mycotoxin, soil, and NDVI values from the 2015 survey. (CSV)

S2 Table Compiled table of maize samples’ associated locations, mycotoxin concentrations, soil, and NDVI values from the 2019 survey. (CSV)

## References

[1] IARC. Mycotoxin control in low- and middle-income countries. Wild CP, Miller DJ, Groopman JD, editors. Geneva, Switzerland; 2008. doi: 10.2514/6.2008-1946 27030861

[2] Mueller DS, Wise KA, Sisson AJ, Allen TW, Bergstrom GC, Bosley DB, et al. Corn yield loss estimates due to diseases in the United States and Ontario, Canada from 2012 to 2015. Plant Health Prog. 2016;17: 211–222. doi: 10.1094/PHP-RS-16-0030

[3] Eskola M, Kos G, Elliott CT, Hajšlová J, Mayar S, Krska R. Worldwide contamination of food-crops with mycotoxins: Validity of the widely cited ‘FAO estimate’ of 25%. Crit Rev Food Sci Nutr. 2019; 1–17. doi: 10.1080/10408398.2019.1658570 31478403

[4] CAST. Mycotoxins: Risks in plant, animal, and human systems. Task Force report, no. 139. Potential Economic Costs of Mycotoxins in the United States. 2003. Available: http://www.cast-science.org/publications/?mycotoxins_risks_in_plant_animal_and_human_systems&show=product&productID=2905

[5] Ross RK, Yu MC, Henderson BE, Yuan JM, Qian GS, Tu JT, et al. Urinary aflatoxin biomarkers and risk of hepatocellular carcinoma. The Lancet. 1992;339: 943–946. doi: 10.1016/0140-6736(92)91528-g 1348796

[6] Williams JH, Phillips TD, Jolly PE, Stiles JK, Jolly CM, Aggarwal D. Human aflatoxicosis in developing countries: A review of toxicology, exposure, potential health consequences, and interventions. Am J Clin Nutr. 2004;80: 1106–1122. doi: 10.1093/ajcn/80.5.1106 15531656

[7] Azziz-Baumgartner E, Lindblade K, Gieseker K, Rogers HS, Kieszak S, Njapau H, et al. Case-control study of an acute aflatoxicosis outbreak, Kenya, 2004. Environ Health Perspect. 2005;113: 1779–1783. doi: 10.1289/ehp.8384 16330363 PMC1314920

[8] Wild CP. Aflatoxin exposure in developing countries: The critical interface of agriculture and health. Food Nutr Bull. 2007;28. doi: 10.1177/15648265070282S217 17658084

[9] Shephard GS. Impact of mycotoxins on human health in developing countries. Food Addit Contam—Part Chem Anal Control Expo Risk Assess. 2008;25: 146–151. doi: 10.1080/02652030701567442 18286404

[10] Kimanya ME, De Meulenaer B, Roberfroid D, Lachat C, Kolsteren P. Fumonisin exposure through maize in complementary foods is inversely associated with linear growth of infants in Tanzania. Mol Nutr Food Res. 2010;54: 1659–1667. doi: 10.1002/mnfr.200900483 20521269

[11] Kamala A, Shirima C, Jani B, Bakari M, Sillo H, Rusibamayila N, et al. Outbreak of an acute aflatoxicosis in Tanzania during 2016. World Mycotoxin J. 2018;11: 311–320. doi: 10.3920/WMJ2018.2344

[12] Turner PC, Collinson AC, Cheung YB, Gong Y, Hall AJ, Prentice AM, et al. Aflatoxin exposure in utero causes growth faltering in Gambian infants. Int J Epidemiol. 2007;36: 1119–1125. doi: 10.1093/ije/dym122 17576701

[13] Cochrane N, D’Souza A. Measuring Access to Food in Tanzania: A Food Basket Approach. Econ Inf Bull. 2015 pp. 1–27.

[14] Ekpa O. Improvement of Maize-Based Foods in Sub-Saharan Africa. Wageningen University. 2020. Available: https://www.proquest.com/docview/2564080514?pq-origsite=gscholar&fromopenview=true&sourcetype=Dissertations & Theses

[15] Mollay C, Kassim N, Stoltzfus R, Kimanya M. Complementary feeding in Kongwa, Tanzania: Findings to inform a mycotoxin mitigation trial. Matern Child Nutr. 2021;17: 1–10. doi: 10.1111/mcn.13188 33945210 PMC8476443

[16] Ngure FM, Kassim N, Phillips EL, Turner PC. Infant and Young Child Feeding Practices and Mycotoxins Contamination of Complementary Food Ingredients in Kongwa District, Tanzania. Curr Dev Nutr. 2023; 100030. doi: 10.1016/j.cdnut.2023.100030 37180082 PMC10111587

[17] Munkvold GP. Epidemiology of Fusarium Diseases and their Mycotoxins in Maize Ears. Eur J Plant Pathol. 2003;109: 705–713. doi: 10.1023/A:1026078324268

[18] Cotty PJ, Mellon JE. Ecology of aflatoxin producing fungi and biocontrol of aflatoxin contamination. Mycotoxin Res. 2006;22: 110–117. doi: 10.1007/BF02956774 23605583

[19] IARC Working Group on the Evaluation of Carcinogenic Risks to Humans. Some traditional herbal medicines, some mycotoxins, naphtalene, and styrene. IARC Monogr Eval Carcinog Risks Hum. Lyon, France: IARC; 2002. PMC4781602 12687954

[20] Bandyopadhyay R, Kumar M, Leslie J. Relative severity of aflatoxin contamination of cereal crops in West Africa. Food Addit Contam. 2007;24: 1109–1114. doi: 10.1080/02652030701553251 17886182

[21] Akello J, Ortega-Beltran A, Katati B, Atehnkeng J, Augusto J, Mwila CM, et al. Prevalence of aflatoxin-and fumonisin-producing fungi associated with cereal crops grown in zimbabwe and their associated risks in a climate change scenario. Foods. 2021;10: 1–18. doi: 10.3390/foods10020287 33572636 PMC7912306

[22] Fandohan P, Zoumenou D, Hounhouigan DJ, Marasas WFO, Wingfield MJ, Hell K. Fate of aflatoxins and fumonisins during the processing of maize into food products in Benin. Int J Food Microbiol. 2005;98: 249–259. doi: 10.1016/j.ijfoodmicro.2004.07.007 15698686

[23] Kimanya ME, De Meulenaer B, Tiisekwa B, Ndomondo-Sigonda M, Devlieghere F, Van Camp J, et al. Co-occurrence of fumonisins with aflatoxins in home-stored maize for human consumption in rural villages of Tanzania. Food Addit Contam—Part Chem Anal Control Expo Risk Assess. 2008;25: 1353–1364. doi: 10.1080/02652030802112601 19680843

[24] Ekpa O, Palacios-Rojas N, Kruseman G, Fogliano V, Linnemann AR. Sub-Saharan African Maize-Based Foods—Processing Practices, Challenges and Opportunities. Food Rev Int. 2019;35: 609–639. doi: 10.1080/87559129.2019.1588290

[25] Matumba L, Van Poucke C, Njumbe Ediage E, De Saeger S. Keeping mycotoxins away from the food: Does the existence of regulations have any impact in Africa? Crit Rev Food Sci Nutr. 2017;57: 1584–1592. doi: 10.1080/10408398.2014.993021 25898143

[26] Jayne TS, Mather D, Mghenyi E. Principal Challenges Confronting Smallholder Agriculture in Sub-Saharan Africa. World Dev. 2010;38: 1384–1398. doi: 10.1016/j.worlddev.2010.06.002

[27] Morton JF. The impact of climate change on smallholder and subsistence agriculture. Proc Natl Acad Sci U S A. 2007;104: 19680–19685. doi: 10.1073/pnas.0701855104 18077400 PMC2148357

[28] Jones PG, Thornton PK. The potential impacts of climate change on maize production in Africa and Latin America in 2055. Glob Environ Change. 2003;13: 51–59. doi: 10.1016/S0959-3780(02)00090-0

[29] Challinor A, Wheeler T, Garforth C, Craufurd P, Kassam A. Assessing the vulnerability of food crop systems in Africa to climate change. Clim Change. 2007;83: 381–399. doi: 10.1007/s10584-007-9249-0

[30] Ahmadalipour A, Moradkhani H, Castelletti A, Magliocca N. Future drought risk in Africa: Integrating vulnerability, climate change, and population growth. Sci Total Environ. 2019;662: 672–686. doi: 10.1016/j.scitotenv.2019.01.278 30703725

[31] Warnatzsch EA, Reay DS, Camardo Leggieri M, Battilani P. Climate Change Impact on Aflatoxin Contamination Risk in Malawi’s Maize Crops. Front Sustain Food Syst. 2020;4: 1–13. doi: 10.3389/fsufs.2020.591792

[32] Alberts J, Rheeder J, Gelderblom W, Shephard G, Burger HM. Rural subsistence maize farming in South Africa: Risk assessment and intervention models for reduction of exposure to fumonisin mycotoxins. Toxins. 2019;11: 1–20. doi: 10.3390/toxins11060334 31212811 PMC6628387

[33] Kebede H, Abbas HK, Fisher DK, Bellaloui N. Relationship between aflatoxin contamination and physiological responses of corn plants under drought and heat stress. Toxins. 2012;4: 1385–1403. doi: 10.3390/toxins4111385 23202322 PMC3509714

[34] Vaughan MM, Huffaker A, Schmelz EA, Dafoe NJ, Christensen SA, McAuslane HJ, et al. Interactive effects of elevated [CO2] and drought on the maize phytochemical defense response against mycotoxigenic Fusarium verticillioides. PLoS ONE. 2016;11: 1–24. doi: 10.1371/journal.pone.0159270 27410032 PMC4943682

[35] Battilani P, Camardo Leggieri M, Rossi V, Giorni P. AFLA-maize, a mechanistic model for Aspergillus flavus infection and aflatoxin B1 contamination in maize. Comput Electron Agric. 2013. doi: 10.1016/j.compag.2013.03.005

[36] Chauhan Y, Tatnell J, Krosch S, Karanja J, Gnonlonfin B, Wanjuki I, et al. An improved simulation model to predict pre-harvest aflatoxin risk in maize. Field Crops Res. 2015;178: 91–99. doi: 10.1016/j.fcr.2015.03.024

[37] Janse van Rensburg B, McLaren NW, Flett BC. Grain colonization by fumonisin-producing Fusarium spp. and fumonisin synthesis in South African commercial maize in relation to prevailing weather conditions. Crop Prot. 2017;102: 129–136. doi: 10.1016/j.cropro.2017.08.019

[38] Cotty PJ, Jaime-Garcia R. Influences of climate on aflatoxin producing fungi and aflatoxin contamination. Int J Food Microbiol. 2007;119: 109–115. doi: 10.1016/j.ijfoodmicro.2007.07.060 17881074

[39] Cao A, Santiago R, Ramos AJ, Souto XC, Aguín O, Malvar RA, et al. Critical environmental and genotypic factors for Fusarium verticillioides infection, fungal growth and fumonisin contamination in maize grown in northwestern Spain. Int J Food Microbiol. 2014;177: 63–71. doi: 10.1016/j.ijfoodmicro.2014.02.004 24607861

[40] Spoto F, Sy O, Laberinti P, Martimort P, Fernandez V, Colin O, et al. Overview of Sentinel-2. IEEE International Geoscience and Remote Sensing Symposium. Munich, Germany: Institute of Electrical and Electronics Engineers; 2012. pp. 1707–1710. doi: 10.1109/IGARSS.2012.6351195

[41] Hengl T, De Jesus JM, Heuvelink GBM, Gonzalez MR, Kilibarda M, Blagotić A, et al. SoilGrids250m: Global gridded soil information based on machine learning. PLoS ONE. 2017;12. doi: 10.1371/journal.pone.0169748 28207752 PMC5313206

[42] McNally A, Arsenault K, Kumar S, Shukla S, Peterson P, Wang S, et al. A land data assimilation system for sub-Saharan Africa food and water security applications. Sci Data. 2017. doi: 10.1038/sdata.2017.12 28195575 PMC5308203

[43] Maxwell AE, Warner TA, Fang F. Implementation of machine-learning classification in remote sensing: An applied review. Int J Remote Sens. 2018;39: 2784–2817. doi: 10.1080/01431161.2018.1433343

[44] Kerry R, Ingram BR, Navarro F, Ortiz BV, Scully BT. Determining Corn Aflatoxin Risk within Counties in Southern Georgia, USA using Remotely Sensed Data. Adv Anim Biosci. 2017;8: 640–644. doi: 10.1017/s2040470017000565

[45] Boken VK, Hoogenboom G, Williams JH, Diarra B, Dione S, Easson GL. Monitoring peanut contamination in Mali (Africa) using AVHRR satellite data and a crop simulation model. Int J Remote Sens. 2008;29: 117–129. doi: 10.1080/01431160701264250

[46] Smith L, Stasiewicz M, Hestrin R, Morales L, Mutiga S, Nelson R. Examining environmental drivers of spatial variability of aflatoxin accumulation in Kenyan maize: potential utility in risk prediction models. Afr J Food Agric Nutr Dev. 2016;3: 11086–11105.

[47] Schaafsma AW, Hooker DC. Climatic models to predict occurrence of Fusarium toxins in wheat and maize. Int J Food Microbiol. 2007;119: 116–125. doi: 10.1016/j.ijfoodmicro.2007.08.006 17900733

[48] Damianidis D, Ortiz BV, Windham GL, Bowen KL, Hoogenboom G, Scully BT, et al. Evaluating a generic drought index as a predictive tool for aflatoxin contamination of corn: From plot to regional level. Crop Prot. 2018;113: 64–74. doi: 10.1016/j.cropro.2018.07.013

[49] Chauhan YS, Wright GC, Rachaputi NC. Modelling climatic risks of aflatoxin contamination in maize. Aust J Exp Agric. 2008;48: 358–366. doi: 10.1071/EA06101

[50] Thomas TS, Robertson R, Boote K. Evaluating risk of aflatoxin field contamination from climate change using new modules inside DSSAT. International Food Policy Research Institute; 2019.

[51] Hell K, Fandohan P, Bandyopadhyay R, Kiewnick S, Sikora R, Cotty. Pre- and Postharvest Management of Aflatoxin in Maize: An African Perspective. In: Leslie JF, Bandyopadhyay R, Visconti A, editors. Mycotoxins: Detection methods, management, public health and agricultural trade. CABI; 2008. pp. 219–229. doi: dx.doi.org/10.1079/9781845930820.0309

[52] Battilani P, Pietri A, Barbano C, Scandolara A, Bertuzzi T, Marocco A. Logistic regression modeling of cropping systems to predict fumonisin contamination in maize. J Agric Food Chem. 2008;56: 10433–10438. doi: 10.1021/jf801809d 18841987

[53] Mutiga SK, Hoffmann V, Harvey JW, Milgroom MG, Nelson RJ. Assessment of Aflatoxin and Fumonisin Contamination of Maize in Western Kenya. Phytopathology. 2015. doi: 10.1094/PHYTO-10-14-0269-R 25894319

[54] Afolabi CG, Bandyopadhyay R, Leslie JF, Ekpo EJA. Effect of Sorting on Incidence and Occurrence of Fumonisins and Fusarium verticillioides on Maize from Nigeria. J Food Prot. 2006;69: 2019–2023. doi: 10.4315/0362-028x-69.8.2019 16924936

[55] Mutiga SK, Were V, Hoffmann V, Harvey JW, Milgroom MG, Nelson RJ. Extent and drivers of mycotoxin contamination: inferences from a survey of Kenyan maize mills. Phytopathology. 2014;104: 1221–1231. doi: 10.1094/PHYTO-01-14-0006-R 24835222

[56] Fandohan P, Ahouansou R, Houssou P, Hell K, Marasas WFO, Wingfield MJ. Impact of mechanical shelling and dehulling on Fusarium infection and fumonisin contamination in maize. Food Addit Contam. 2006;23: 415–421. doi: 10.1080/02652030500442516 16546888

[57] Shu X, Livingston DP, Woloshuk CP, Payne GA. Comparative Histological and Transcriptional Analysis of Maize Kernels Infected with Aspergillus flavus and Fusarium verticillioides. Front Plant Sci. 2017;8: 1–14. doi: 10.1097/00006247-197708000-00005 29270183 PMC5723656

[58] Ohna I, Kaarhus R, Kinabo J. No Meal without Ugali? Social Significance of Food and Consumption in a Tanzanian Village. Cult Agric Food Environ. 2012;34: 3–14. doi: 10.1111/j.2153-9561.2012.01061.x

[59] Harvey J, Gnonlonfin B, Fletcher M, Fox G, Trowell S, Berna A, et al. Improving diagnostics for aflatoxin detection. In: Unnevehr L, Grace D, editors. Aflatoxins: Finding Solutions for Improved Food Safety. 2013. doi: 10.2499/9780896296763

[60] Pearson TC, Wicklow DT, Pasikatan MC. Reduction of aflatoxin and fumonisin contamination in yellow corn by high-speed dual-wavelength sorting. Cereal Chem. 2004. doi: 10.1094/CCHEM.2004.81.4.490

[61] Stasiewicz MJ, Falade TDO, Mutuma M, Mutiga SK, Harvey JJW, Fox G, et al. Multi-spectral kernel sorting to reduce aflatoxins and fumonisins in Kenyan maize. Food Control. 2017;78: 203–214. doi: 10.1016/j.foodcont.2017.02.038

[62] Aoun M, Siegel C, Windham GL, Williams WP, Nelson RJ. Application of reflectance spectroscopy to identify maize genotypes and aflatoxin levels in single kernels. World Mycotoxin J. 2022;15: 1–16. doi: 10.3920/wmj2021.2750

[63] Temba BA, Darnell RE, Gichangi A, Lwezaura D, Pardey PG, Harvey JJ, et al. The influence of weather on the occurrence of aflatoxin B1 in harvested maize from Kenya and Tanzania. Foods. 2021;10. doi: 10.3390/foods10020216 33494323 PMC7911846

[64] Director KDE. Kongwa District: Social-Economic Profile. 2016.

[65] Phillips E, Ngure F, Smith LE, Makule E, Turner PC, Nelson R, et al. Protocol for the trial to establish a causal linkage between mycotoxin exposure and child stunting: A cluster randomized trial. BMC Public Health. 2020;20: 1–11. doi: 10.1186/s12889-020-08694-6 32357944 PMC7193337

[66] Cooper D, Callahan B, Callahan P, Burnett L. Mobile Image Ratiometry: A New Method for Instantaneous Analysis of Rapid Test Strips. Nat Preced. 2012; 2–3. doi: 10.1038/npre.2012.6827.1

[67] Hollister J, Shah T, Robitaille AL, Beck MW, Johnson M. elevatr: Access Elevation Data from Various APIs. 2021. doi: 10.5281/zenodo.5809645

[68] R Core Team. R: A Language and Environment for Statistical Computing. Vienna, Austria: R Foundation for Statistical Computing; 2022. Available: https://www.r-project.org/

[69] Stevens A, Ramirez-Lopez L. An introduction to the prospectr package. 2022. Available: https://cran.r-project.org/web/packages/prospectr/vignettes/prospectr.html

[70] Hijmans RJ. raster: Geographic Data Analysis and Modeling. 2023. Available: https://cran.r-project.org/package=raster

[71] Bates D, Mächler M, Bolker B, Walker S. Fitting Linear Mixed-Effects Models Using {lme4}. J Stat Softw. 2015;67: 1–48. doi: 10.18637/jss.v067.i01

[72] Kuznetsova A, Brockhoff PB, Christensen RHB. {lmerTest} Package: Tests in Linear Mixed Effects Models. J Stat Softw. 2017;82: 1–26. doi: 10.18637/jss.v082.i13

[73] Bartoń K. MuMIn: Multi-Model Inference. 2022. Available: https://cran.r-project.org/package=MuMIn

[74] Liaw A, Wiener M. Classification and Regression by randomForest. R News. 2002;2: 18–22.

[75] Wickham H. ggplot2: Elegant Graphics for Data Analysis. New York: Springer-Verlag; 2016. Available: https://ggplot2.tidyverse.org

[76] Kassambara A. ggpubr: “ggplot2” Based Publication Ready Plots. 2023. Available: https://cran.r-project.org/package=ggpubr

[77] EAC. East African Standard Maize Grains Specification. 2013 pp. 1–18.

[78] Ng’ambi JT, Atehnkeng J, Monjerezi M, Ngongondo C, Vunain E, Ching’anda C, et al. Micro-climatic variations across Malawi have a greater influence on contamination of maize with aflatoxins than with fumonisins. Mycotoxin Res. 2022. doi: 10.1007/s12550-022-00471-1 36443622 PMC10156841

[79] Kosmowski F, Worku T. Evaluation of a miniaturized NIR spectrometer for cultivar identification: The case of barley, chickpea and sorghum in Ethiopia. PLoS ONE. 2018;13: 1–17. doi: 10.1371/journal.pone.0193620 29561868 PMC5862431

[80] Riu J, Gorla G, Chakif D, Boqué R, Giussani B. Rapid analysis of milk using low-cost pocket-size NIR spectrometers and multivariate analysis. Foods. 2020;9. doi: 10.3390/foods9081090 32785190 PMC7465951

[81] Hershberger J, Mbanjo EGN, Peteti P, Ikpan A, Ogunpaimo K, Nafiu K, et al. Low-cost, handheld near-infrared spectroscopy for root dry matter content prediction in cassava. Plant Phenome J. 2022;5. doi: 10.1002/ppj2.20040

[82] Lewis L, Onsongo M, Njapau H, Schurz-Rogers H, Luber G, Kieszak S, et al. Aflatoxin contamination of commercial maize products during an outbreak of acute aflatoxicosis in eastern and central Kenya. Environ Health Perspect. 2005;113: 1763–1767. doi: 10.1289/ehp.7998 16330360 PMC1314917

[83] Daniel JH, Lewis LW, Redwood YA, Kieszak S, Breiman RF, Dana flanders W, et al. Comprehensive assessment of maize aflatoxin levels in eastern Kenya, 2005–2007. Environ Health Perspect. 2011;119: 1794–1799. doi: 10.1289/ehp.1003044 21843999 PMC3261970

[84] Hoffman V, Mutiga S, Harvey J, Nelson RJ, Milgroom M. Asymmetric information and food safety: Maize in Kenya. Agricultural & Applied Economics Association’s 2013 AAEA & CAES Joint Annual Meeting. Washington, DC; 2013. Available: https://ageconsearch.umn.edu/record/151288/

[85] Hell K, Cardwell KF, Setamou M, Poehling HM. The influence of storage practices on aflatoxin contamination in maize in four agroecological zones of Benin, West Africa. J Stored Prod Res. 2000;36: 365–382. doi: 10.1016/s0022-474x(99)00056-9 10880814

[86] Probst C, Callicott KA, Cotty PJ. Deadly strains of Kenyan Aspergillus are distinct from other aflatoxin producers. Eur J Plant Pathol. 2012;132: 419–429. doi: 10.1007/s10658-011-9887-y

[87] Wenndt AJ, Sudini HK, Mehta R, Pingali P, Nelson R. Spatiotemporal assessment of post-harvest mycotoxin contamination in rural North Indian food systems. Food Control. 2021;126. doi: 10.1016/j.foodcont.2021.108071 34345120 PMC8075802

[88] Stafstrom W, Wenndt A, Nelson R. Mycotoxin Surveillance for Low-resource Settings. Mycotoxins in Food and Beverages Innovations and Advances Part I. 2021. pp. 1–29. doi: 10.1201/9781003035817-1

[89] Mrisho LM, Mbilinyi NA, Ndalahwa M, Ramcharan AM, Kehs AK, McCloskey PC, et al. Accuracy of a Smartphone-Based Object Detection Model, PlantVillage Nuru, in Identifying the Foliar Symptoms of the Viral Diseases of Cassava–CMD and CBSD. Front Plant Sci. 2020;11: 1–14. doi: 10.3389/fpls.2020.590889 33391304 PMC7775399

[90] Radhakrishnan GV, Cook NM, Bueno-Sancho V, Lewis CM, Persoons A, Mitiku AD, et al. MARPLE, a point-of-care, strain-level disease diagnostics and surveillance tool for complex fungal pathogens. BMC Biol. 2019. doi: 10.1186/s12915-019-0684-y 31405370 PMC6691556

