# Supplementary material for: Modeling maize aflatoxins and fumonisins in a Tanzanian smallholder system: Accounting for diverse risk factors improves mycotoxin models
Source: PLoS One. 2025 Jan 13;20(1):e0316457. doi: 10.1371/journal.pone.0316457 (PMC11729969; doi:10.1371/journal.pone.0316457)
Supplement: S1 Appendix — (DOCX) [file pone.0316457.s001.docx]

|  | Questions (English) | Maswali (Kiswahili) |
| --- | --- | --- |
| 1 | Which village is the maize farm in? | Shamba la mahindi lipo kwenye kijiji gani? |
| 2 | How long does it take (minutes) to walk from farm to mill? | Unatumia muda gani kufika kwenye mashine kutoka shambani kwako (dakika)? |
| 3 | When was this maize planted? | Mahindi haya yalipandwa lini? |
| 4 | When was this maize harvested? | Mahindi haya yalivunwa lini? |
| 5 | What type of maize seed is this? | Hii mbegu ya mhindi ni ya aina gani? |
| 5a* | Other type of seed? | Aina nyingine ya mbegu? |
| 6 | Was the maize planted alone or intercropped? | Je ulipanda mahindi tu au ulichanganya na zao jingine? |
| 6a* | Which crop was it intercropped with? | Ulipanda zao gani pamoja na mahindi? |
| 7 | Did you use fertilizer on your maize? | Je ulitumia mbolea kwenye mahindi haya, na ilikuwa ya aina gani? |
| 8 | Was there water (drought) stress during this crop’s growth? | Kulikuwa na uhaba wa mvua wakati mahindi yanakua? |
| 8a* | When was the maize most affected by water stress/ lack of rain? | Je ni kipindi gani mahindi yaliathiriwa sana na uhaba wa mvua? |
| 9 | Would you describe the insect/pest problems as normal, better than normal, or worse than normal? | Elezea kipimo cha tatizo la wadudu au magonjwa kuvamia mahindi: kawaida, mbaya kuliko kawaida, nzuri kuliko kawaida? |
| 10 | At the time of harvest was the maize fully matured? | Je wakati unavuna, mahindi yalikuwa yameshakomaa kabisa? |
| 10a* | Was the matured maize rained on before harvesting? | Je, haya mahindi yaliyokomaa yalinyeshewa na mvua kabla ya kuvuna? |
| 11 | At the time of harvest had the maize dried adequately/completely? | Je wakati wa kuvuna, mahindi yalikuwa yamekauka kabisa? |
| 11a* | Was the adequately dried maize rained on before harvesting? | Je mvua iliyanyeshea haya mahindi makavu kabla ya kuvuna? |
| 12 | After harvesting, where did you dry the maize? | Ulikausha wapi mahindi baada ya kuvuna? |
| 12a* | Where was it dried? | Ulikausha wapi? |
| 13 | Did you shell the maize before drying? | Ulipukuchua haya mahindi kabla ya kukauka? |
| 14 | How was this maize stored after drying? | Mahindi yalihifadhiwa wapi baada ya kukauka? |
| 14a* | Other storage type? | Aina nyingine ya kuhifadhi? |
| 15 | When and how was this maize sorted? | Mahindi haya yalichambuliwa/pepetwa wakati gani? |
| 15a* | How was it sorted? | Ulichambuaje/ulipepetaje? |
| 16 | Which type of maize flour is this? | Unga huu wa mahindi ni wa aina gani? |

*Dependent on previous question’s answer.
